# Novel Strategies to Inhibit Pertussis Toxin

**DOI:** 10.3390/toxins14030187

**Published:** 2022-03-03

**Authors:** Katharina Ernst

**Affiliations:** Institute of Pharmacology and Toxicology, Ulm University Medical Center, 89081 Ulm, Germany; katharina.ernst@uni-ulm.de

**Keywords:** pertussis toxin, novel inhibitors, chaperones, humanized antibodies, human defensins, ADP-ribosylation inhibitor

## Abstract

Pertussis, also known as whooping cough, is a respiratory disease caused by infection with *Bordetella pertussis*, which releases several virulence factors, including the AB-type pertussis toxin (PT). The characteristic symptom is severe, long-lasting paroxysmal coughing. Especially in newborns and infants, pertussis symptoms, such as leukocytosis, can become life-threatening. Despite an available vaccination, increasing case numbers have been reported worldwide, including Western countries such as Germany and the USA. Antibiotic treatment is available and important to prevent further transmission. However, antibiotics only reduce symptoms if administered in early stages, which rarely occurs due to a late diagnosis. Thus, no causative treatments against symptoms of whooping cough are currently available. The AB-type protein toxin PT is a main virulence factor and consists of a binding subunit that facilitates transport of an enzyme subunit into the cytosol of target cells. There, the enzyme subunit ADP-ribosylates inhibitory α-subunits of G-protein coupled receptors resulting in disturbed cAMP signaling. As an important virulence factor associated with severe symptoms, such as leukocytosis, and poor outcomes, PT represents an attractive drug target to develop novel therapeutic strategies. In this review, chaperone inhibitors, human peptides, small molecule inhibitors, and humanized antibodies are discussed as novel strategies to inhibit PT.

## 1. Introduction

Whooping cough is highly contagious and is caused by droplet infection of the upper respiratory tract with *Bordetella (B.) pertussis*. This bacterial pathogen produces several virulence factors that contribute to the development of the disease, including the AB-type pertussis toxin (PT) [1]. The characteristic paroxysmal coughing that causes the patient to make a “whooping” sound upon inhalation between coughing attacks, typically lasts for up to 10 weeks. *B. pertussis* infection leads to potent inflammatory responses that cause severe lung damage [2,3]. Especially, newborns and infants are vulnerable to complications such as pneumonia, encephalopathy, seizures or apnea that occur in severe cases and can be life-threatening [4]. In Germany, for instance, 53% of infants suffering from pertussis had to be hospitalized due to severe course of disease in 2018 [5]. Worldwide, over 24.1 million pertussis cases and 160,000 deaths have been estimated by the WHO in 2014 in children under 5 years of age [6]. Pertussis was described as the leading cause of vaccine-preventable deaths and morbidity globally [7].

For successful colonization of the ciliated epithelia of the human respiratory tract, *B. pertussis* requires adhesion factors, such as filamentous hemagglutinin (FHA) and fimbriae [1]. Other toxins, beside PT, that alter physiological functions are the tracheal cytotoxin, which targets and kills ciliated cells and the adenylate cyclase toxin, which inhibits phagocytosis by excessive cAMP generation in phagocytes, thereby protecting the bacteria from elimination by the immune system [8,9]. The precise role of PT and other virulence factors, as well as their interplay for the *B. pertussis* pathogenesis, have not been elucidated in detail. For example, it is not fully understood how the severe coughing pathology is caused. Administration of purified PT to experimental animals does not elicit the cough pathology of pertussis. It is assumed that the cough pathology is a product of complex host responses to infection combined with the activity of the released toxins [10,11]. Severe and long-lasting inflammation of the airways was caused by PT in a mouse model [12]. Leukocytosis is a PT-associated characteristic hallmark of severe pertussis and correlates with poor prognosis [10,13]. Leukocytosis and death were associated with expression of PT since strains lacking PT only caused mild disease symptoms and did not result in leukocytosis or death [10,13,14,15,16]. This clearly demonstrates the crucial role of PT in causing severe courses of disease.

Worldwide, a relatively high vaccination coverage of ca. 85% of infants was estimated in 2019, with even higher coverage in Western countries (e.g., in Germany ca. 93% of children between 5–7 years of age were vaccinated in 2017 [17,18]). Despite these high vaccination rates, increasing case numbers have been recorded in Western countries, reaching an all-time high since the introduction of pertussis vaccination in the 1950s [19]. The first vaccines used were based on inactivated whole-cell *B. pertussis* preparations. Although causing a striking drop in pertussis incidence, vaccine-associated adverse effects encouraged the development of a more defined acellular pertussis vaccine [20]. All available acellular vaccines contain detoxified PT as the main protective antigen and most of them also contain filamentous hemagglutinin, as well as additional antigens [20].

Most high-income countries have currently replaced the whole-cell with the acellular vaccine, which shows an improved safety profile over the whole-cell vaccine. However, several studies have suggested that the acellular vaccine protects against pertussis disease but does not prevent infection and transmission [20,21]. Moreover, fast waning of vaccine-induced immunity was also reported for the acellular compared to the whole-cell vaccine [22,23]. Genetic adaptation of circulating strains to escape vaccine pressure is also discussed as a reason for resurgence of pertussis despite high vaccination rates [22,24].

The increasing case numbers during the last years clearly indicate that pertussis disease is not under control and on the rise again. This, together with the lack of therapeutic strategies against whooping cough, emphasizes the necessity to find new pharmacological approaches against this toxin-mediated disease. Since PT is crucial for eliciting the disease, it represents an attractive target for the development of novel pharmacological strategies [10,13,25]. In this review, the structure, cellular uptake and mode of action of PT, as well as novel strategies to inhibit PT, are discussed.

## 2. Pertussis Toxin—Structure, Cellular Uptake and Mode of Action

**Figure 1:**
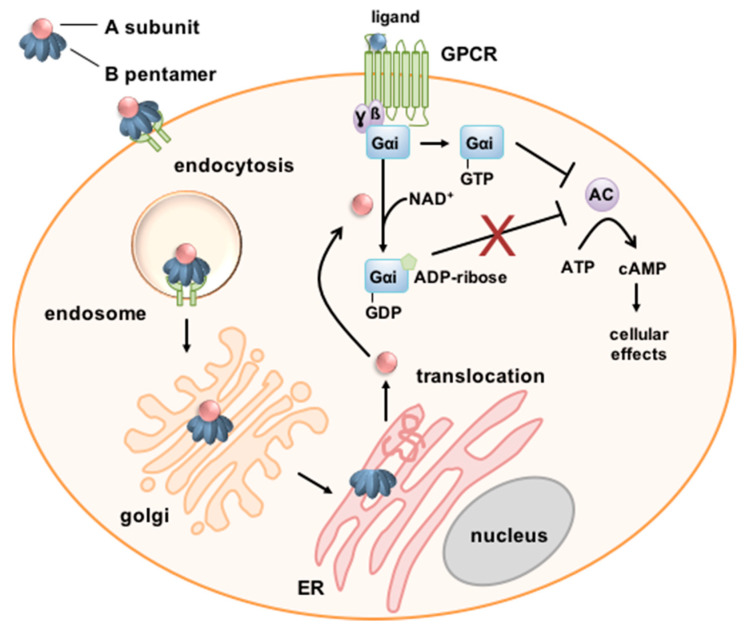
Schematic depiction of the cellular uptake and mode of action of PT. PT binds via its B-subunit to the cell surface. PT is endocytosed and follows a retrograde route through the Golgi to the endoplasmic reticulum (ER). The A-subunit PTS1 dissociates from the B-Oligomer and is translocated via the ER-associated degradation (ERAD) pathway into the cytosol. Here, PTS1 ADP-ribosylates inhibitory α-subunits (Gαi) of G-protein coupled receptors (GPCR) thereby inactivating it. Thus, if the respective GPCR is activated, Gαi is no longer able to inhibit the adenylate cyclase (AC) resulting in increased and disturbed cAMP signaling [28,33].

As an AB_5_ toxin, PT consists of an enzyme subunit, the A-protomer PTS1 (pertussis toxin subunit 1), and four different B-subunits, PTS2, PTS3, PTS4 and PTS5 in a ratio of 1:1:2:1 [26,27] (Figure 1). The B-pentamer consisting of PT subunits 2 to 5 and PTS1 assemble through non-covalent bonds in the periplasm of the bacteria to form a PT holotoxin with a pyramid-like structure. The holotoxin is secreted by a type IV secretion system [26,28]. The B-pentamer binds to the cell surface via sialoglycoproteins that are found on most cells, enabling PT to intoxicate various cell types [29,30,31]. A specific receptor has not been identified so far.

After internalization by endocytosis, PT takes a retrograde intracellular route through the Golgi to the endoplasmic reticulum (ER) [32,33]. In the ER, binding of ATP to the toxin causes destabilization of the interaction between PTS1 and the B-pentamer resulting in release of PTS1 from the holotoxin [34,35,36]. Under physiological conditions, released PTS1 is thermally unstable resulting in its unfolding [37,38,39]. This leads to transport of PTS1 by the ER-associated degradation (ERAD) pathway from the ER to the cytosol. PTS1 lacks lysine residues and is, therefore, protected from ubiquitination and thus from proteasomal degradation [40]. The molecular mechanism of the membrane transport of PTS1 into the cytosol remains to be understood in detail. For example, it is not known how the unfolded PTS1 regains its active conformation in the cytosol. However, a crucial role of cellular protein folding helper enzymes for uptake of PTS1 into the host-cell cytosol has been demonstrated (see Section 3.1) [41,42,43].

In the cytosol, PTS1 mediates the covalent transfer of an ADP-ribose moiety from the co-substrate NAD^+^ onto α-subunits of inhibitory G-proteins (Gαi) [20,44,45]. ADP-ribosylation causes loss of function of Gαi. Therefore, if the corresponding G-protein coupled receptor (GPCR) is activated, Gαi can no longer inhibit the adenylate cyclase, resulting in increased and disturbed cAMP signaling. The disturbed cAMP signaling causes various effects that depend on the cell type. In early stages of infection, recruitment of neutrophils, monocytes and lymphocytes to the lung is inhibited by PT which leads to reduced levels of pro-inflammatory chemokines and cytokines and to increased bacterial burden in a mouse infection model [46,47,48].

## 3. Novel Inhibitors of Pertussis Toxin

**Figure 2:**
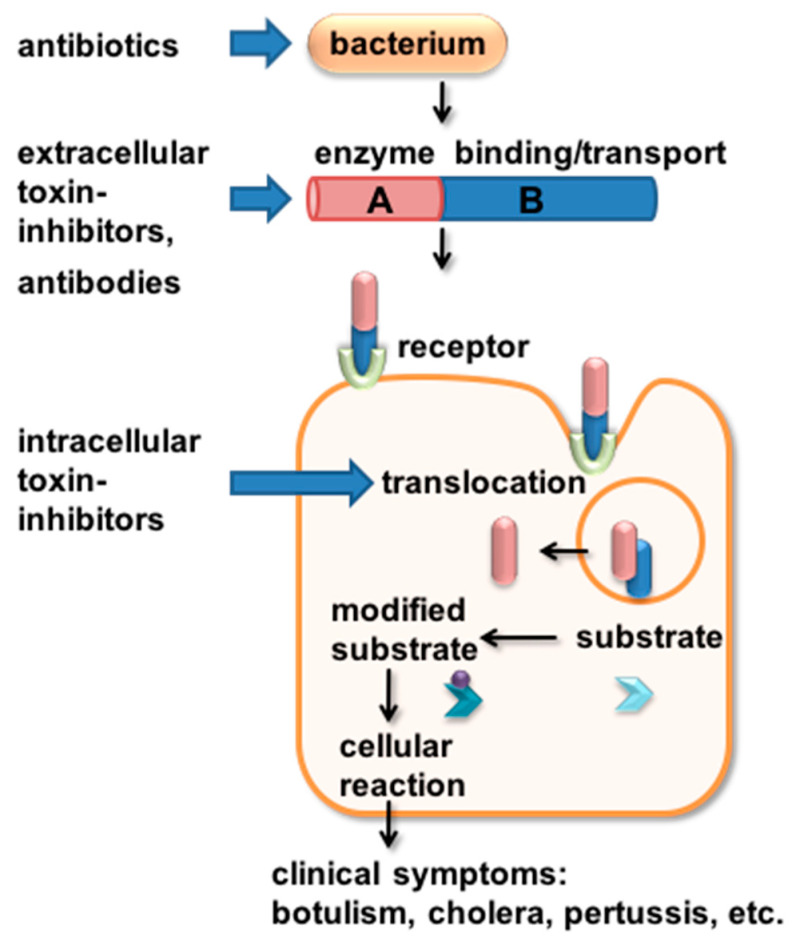
Schematic depiction of different targets of AB-type toxin-inhibitors. AB-type toxins can be targeted in various ways by inhibitors. The toxin-producing bacteria are targeted by antibiotics. The released toxins that act independently of the bacteria can be targeted extracellularly by neutralizing antibodies or peptides. If the toxin already entered the cell, small molecule inhibitors of toxin enzyme activity or of cellular proteins that aid the translocation of the toxin enzyme subunit into the cytosol still can protect the target cell from intoxication. Details are given in the text.

Therapeutic options to treat pertussis are very limited. Antibiotic therapy eliminates *B. pertussis* bacteria, which is important to prevent further transmission by droplet infection (Figure 2) [7,49]. The macrolides azithromycin, clarithromycin and erythromycin are mainly used; however, antibiotic treatment has no relieving effect on pertussis symptoms except if treatment is started within two weeks after infection, which rarely occurs because in most cases the diagnosis is made later [4,50].

Increasing case numbers during the last few years and the lack of treatment options require the development of novel therapeutic strategies to treat pertussis. Since whooping cough is a toxin-mediated disease and PT is the central factor that promotes severity of the disease, PT is a promising target for development of novel pharmacological strategies against severe pertussis in infants, and also against cough symptomology in older patients with pertussis.

Inhibitors can affect the toxin in different ways (Figure 2). Neutralizing inhibitors such as antibodies (see Section 3.4) or peptides (see Section 3.2) interact or bind the toxin before or during receptor-binding. This prevents the toxin from entering its target cell. Once taken up into the cell by receptor-mediated endocytosis, antibodies can no longer reach and neutralize the toxin. Inhibitors interfering with later steps of toxin uptake such as translocation into the cytosol (see Section 3.1) or enzyme activity (see Section 3.3) provide the opportunity to inhibit toxin molecules already taken up by cells. An overview of the inhibitors described in this review is given in Table 1.

### 3.1. Pharmacological Chaperone Inhibitors

During the last few years, we investigated and characterized the cellular uptake mechanism of ADP-ribosylating toxins, including PT, with a focus on the ability of these toxins to translocate their enzyme subunits across intracellular membranes to escape vesicular compartments and reach the cytosol of their target cells [57,58]. This step is crucial for the pathogenic mode of action because the specific substrate of these toxins resides in the cytosol. If the translocation into the cytosol is inhibited, the cytotoxic effect and thus the clinical symptoms caused by the toxins can be prevented. Therefore, it was demonstrated over the last years that intracellular membrane translocation of these toxins represents an attractive starting point for novel pharmacological strategies. Pharmacological inhibitors of intracellular membrane transport are able to inhibit the enzyme subunits of bacterial toxins even when they have already been endocytosed. Therefore, these inhibitors have the potential to complement therapeutic strategies aiming at neutralizing toxins, e.g., human peptides or antibodies that interact with the toxin before it binds or enters cells (Figure 2).

**Figure 3:**
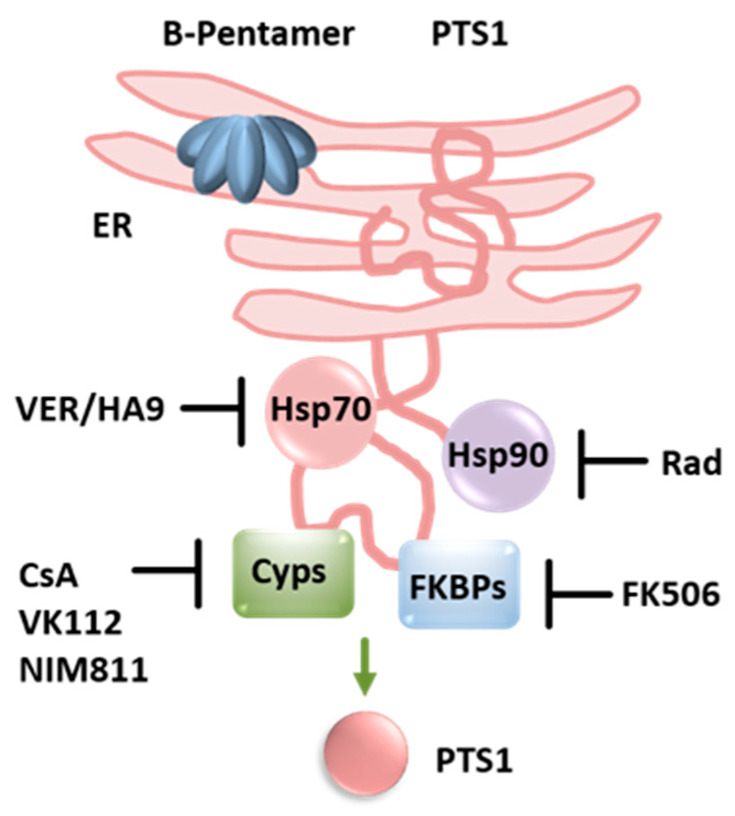
Proposed mechanism for mode of action of chaperones during uptake of PT into cells. PT reaches the ER via a retrograde intracellular transport. ATP binding leads to the release and unfolding of the enzyme subunit PTS1. PTS1 is recognized by the ER-associated degradation pathway and transported into the cytosol. If the activity of chaperones Hsp90 or Hsp70 or peptidyl-prolyl cis/trans isomerases of the cyclophilin or FK506 binding protein family are inhibited by specific pharmacological inhibitors, less PTS1 reaches the cytosol [41,42,43]. (Rad = radicicol, VK112, NIM811 = non-immunosuppressive CsA derivatives; further explanations are given in the text).

To reach the cytosol of target cells, PTS1 dissociates from the B-oligomer in the ER upon ATP binding. The crystal structure of PT associated with ATP revealed that ATP was localized at the area of the B-oligomer that interacts with PTS1 [35]. ATP seems to activate PT by destabilizing the PT holotoxin and facilitating the cleavage of a disulfide bond in PTS1 [59,60], thereby allowing interaction with the substrate [26]. Moreover, it was shown that a PT mutant that shows only a reduced interaction with ATP is less active in vitro and shows reduced ADP-ribosylation activity in cells, as well as reduced dissociation by interaction with ATP [36]. Dissociated PTS1 is thermally unstable and is subsequently recognized as a substrate of the ERAD pathway facilitating its translocation into the cytosol (Figure 1 and Figure 3). Therefore, we hypothesized that intracellular chaperones and protein folding helper enzymes might play a role for uptake and mode of action of PT. We showed that inhibitors of cellular chaperones protect cells from intoxication with PT [41,42,43]. We characterized the underlying mode of inhibition and demonstrated that chaperones of the heat shock family (Hsp90, Hsp70) and of the peptidyl prolyl cis/trans isomerase (PPIase) family (cyclophilins, Cyps and FK506-binding proteins, FKBPs) are required for the transport of the enzyme subunit of PT, as well as other ADP-ribosylating toxins into the target cell cytosol [58,61,62,63,64,65]. Specific pharmacological inhibitors that block the activity of different chaperones prevented transport of enzyme subunits of the clostridial enterotoxins *C. botulinum* C2 toxin, *C. perfringens* iota toxin and *C. difficile* CDT toxin, as well as of *B. pertussis* toxin (Figure 3).

The inhibitor radicicol binds with high affinity to the ATP-binding site of Hsp90, thereby inhibiting its chaperone activity. Hydrolysis of ATP is required to elicit conformational changes in Hsp90 which leads to conformational changes in the client protein, i.e., the protein requiring folding assistance [66,67]. The activity of Hsp70 is also ATP-dependent and can be inhibited by the specific pharmacological inhibitor VER-155008 (VER), which also binds to its ATP-binding site [68]. Another novel Hsp70 inhibitor, HA9, binds to the substrate-binding domain of Hsp70 and thereby also inactivates Hsp70 activity [63]. Cyps and FKBPs catalyze the cis/trans isomerization of proline in peptide bonds, which is a rate-limiting step in protein folding. Cyps and FKBPs are inhibited by Cyclosporine A (CsA) and FK506, respectively, due to binding to their PPIase domain [69,70]. CsA and FK506 are licensed drugs with immunosuppressive effects used for example after organ transplantation to prevent organ rejection. Since chaperones and PPIases are involved in various processes of protein folding, possible off target effects have to be considered in regard to application of pharmacological chaperone inhibitors to inhibit PT or other AB-type toxins. Local instead of systemic application of inhibitors might be a beneficial strategy in this matter.

To investigate activity and/or inhibition of PT in vitro, the cell line CHO (Chinese hamster ovary) is used because these cells show a specific clustering morphology after treatment with purified PT [71,72]. Pre-incubation of CHO cells with either radicicol, VER, HA9, CsA or FK506 resulted in a decreased PT-induced clustering morphology and reduced ADP-ribosylated Gαi by PTS1 in lysates from PT-treated cells [41,42]. CsA and FK506 also prevented PT-mediated effects on cAMP signaling [41].

After establishing a functional role of chaperones/PPIases during PT uptake into cells we investigated the underlying mechanism in more detail. The inhibitors had no effect on binding of PT to cells or its enzyme activity in vitro. Instead, inhibition of chaperones reduced the amount of PTS1 molecules in the cytosol, which results in reduced ADP-ribosylation of Gαi. This suggests that cellular chaperones/PPIases facilitate translocation of PTS1 from the ER to the cytosol [41,42,43]. Interestingly, a comparable mechanism was discovered for other ADP-ribosylating toxins such as C2, iota and CDT toxin, suggesting a common mechanism of inhibition [62,73,74,75].

A protective effect of CsA and a non-immunosuppressive CsA derivative against severe symptoms of pertussis was also shown in a neonatal *B. pertussis* mouse model [41]. This mouse model reproduces the age-dependent effects of severe pertussis disease. Infant mice are more susceptible to lethal infection than adult mice and also develop leukocytosis like humans [76]. Leukocytosis is PT-mediated and correlates with disease severity and lethality [13]. Therefore, this mouse model is relevant with respect to studying pertussis for human disease. In these experiments, CsA and its non-immunosuppressive derivative had no effect on the colony forming units of *B. pertussis* detected in lung homogenates. However, CsA and its derivative significantly reduced leukocytosis (white blood cell count) in infant mice [41]. These results are of particular interest because in context of a bacterial infection the immunosuppressive effect of CsA would be disadvantageous. The CsA derivative, however, still inhibits PPIase activity of Cyps but lacks the immunosuppressive effect. Moreover, CsA is already a licensed drug, which increases its potential as a novel pharmacological approach to combat severe symptoms of whooping cough [41,42,77].

### 3.2. Human Antimicrobial Peptides of the Defensin Family

Recently, the human antimicrobial peptides α-defensin-1 and -5 have been identified as inhibitors of PT activity [51]. Defensins are part of the innate immunity and are characterized as small, cationic and cysteine-rich peptides. Human defensins comprise α- and ß-defensins which mainly differ in patterns of their three intramolecular disulfide bonds. α-defensins are secreted by neutrophils (human neutrophil peptides (HNP) 1–4) or by Paneth cells (human defensins (HD) 5 and 6). Human ß-defensins (HBD) 1–4 are found in several epithelial cells for example of the respiratory and gastrointestinal tract [78].

Due to their cationic and hydrophobic nature, antimicrobial peptides interact with the negatively charged surface of bacteria and form pores into microbial membranes. Inhibition of cell wall synthesis and immunomodulatory effects by antimicrobial peptides have been reported as well [79]. In addition to their effect on bacteria, antimicrobial peptides, in particular members of the defensin family, have been identified as inhibitors of bacterial toxins such as diphtheria toxin or *Clostridioides difficile* toxins [80,81,82,83,84].

We showed that α-defensin-1 and -5 inhibit the enzyme activity of the A-subunit PTS1 in vitro, in a concentration-dependent manner. α-defensin-1 and -5 also resulted in a reduced amount of ADP-ribosylated Gαi in PT-treated cells. This indicates that the α-defensins protected cells from PT-intoxication. Inhibition of PTS1 in vitro and of PT-intoxication of cells by the α-defensins was observed with and without prior pre-incubation. Thus far, the precise mode of inhibition and interaction of PTS1 with the α-defensins is not fully understood. Blocking of the active site of PTS1 by the α-defensins might be the cause for decreased enzyme activity. Destabilizing or unfolding of the PTS1 protein structure might also be facilitated by the α-defensins, since this was shown for other toxins [84,85].

ADP-ribosylation of Gαi leads to its inactivation and thereby loss of adenylate cyclase inhibition upon receptor stimulation (Figure 1). In a novel living cell-based interference in Gαi-mediated signal transduction (iGIST) assay, the effect of PT on Gαi and thereby cAMP signaling was measured [41,51,86]. This assay is based on HEK293 cells that express the somatostatin receptor 2 (SSTR2), which is a Gαi-protein coupled receptor (GPCR), as well as a luminescent cAMP probe. Direct activation of the adenylate cyclase by forskolin in combination with stimulation of SSTR2 only leads to a moderate increase in cAMP because SSTR2 stimulation results in inhibition of adenylate cyclase. Treatment of cells with PT followed by activation of adenylate cyclase and stimulation of SSTR2 results in high cAMP levels. This is because PTS1 inactivates Gαi of SSTR2 by ADP-ribosylation. α-defensin-1 and -5 both reduced PT-mediated effects on cAMP levels in this novel bioassay indicating that the defensins interfered with PT activity [51].

Unlike α-defensin-1 and -5, ß-defensin-1 had no effect on in vitro enzyme activity, intoxication of cells or PT-mediated effects on cAMP signaling [51]. This suggests a specific inhibition mechanism of α-defensin-1 and -5 on PT activity. The reason why α-defensin-1 and -5 but not ß-defensin-1 inhibit PT is not known yet. α-defensin-1 and -5 have a very similar three-dimensional structure, which differs from the structure of ß-defensin [78,84]. This might be a difference between α-defensin-1 and -5 and ß-defensin-1 determining the specificity in inhibition of PT.

Peptide therapeutics gained increasing importance over the last years with more than 400 peptide drugs currently under clinical developments and over 60 already approved for clinical use in Europe, Japan, and the United States [87]. An advantage of peptides compared to antibodies is for example the ability of peptides to bind into deep folding pockets of target proteins that cannot be reached by antibodies. Moreover, as endogenous agents, peptides are usually better tolerated and less immunogenic than agents based on foreign antigens [87]. However, delivery of peptides across biological membranes is a major challenge and can limit their therapeutic use. Potential off-target effects of defensins have to be explored in future studies and could be addressed by peptide optimization strategies for example regarding their sequence, structure or pharmacokinetic properties. Identification of human peptides such as defensins inhibiting PT and characterization of the underlying mechanism contributes to a better understanding of innate defense mechanisms against PT. Moreover, inhibitory peptides can serve as a starting point for the development of therapeutic strategies against whooping cough.

### 3.3. Small Molecule Inhibitors of PTS1 Enzyme Activity

Recently, Pulliainen and co-workers identified the first small molecule inhibitor of PTS1 enzyme activity by performing a screening of a compound library [52]. Therefore, they first designed a recombinant PTS1 enzyme subunit with N- and C-terminal truncations that allowed efficient expression and purification of active PTS1. This recombinant PTS1 was used in an in vitro high throughput-compatible assay which is based on quantification of NAD^+^ consumption due to PTS1-mediated ADP-ribosylation of Gαi. First, a panel of selected compounds with known inhibitory effects on ADP-ribosylating activity of human ADP-ribosyltransferases was tested which did not yield in any inhibitors against PTS1 activity. Screening of a high scaffold diversity compound library identified seven compounds out of 1695 that inhibited PTS1-mediated NAD^+^ consumption in vitro. Two of these compounds, NSC228155 and NSC29193, showed inhibition of PTS1 with IC_50_ values in the low micromolar range (NSC228155: IC_50_ = 3.0 µM and NSC29193: IC_50_ = 6.8 µM) without affecting protein integrity of either PTS1 or Gαi [52]. The structures of NSC228155 and NSC29193 show similarities to the adenine base or the nicotinamide end of NAD^+^ and docking and molecular dynamics simulations revealed possible binding poses at the PTS1 NAD^+^ binding pocket.

Both compounds were tested regarding their effect on PT-intoxication of living cells (HEK293T cells). Therefore, cells were pre-incubated with NSC228155 or NSC29193 and after subsequent treatment with PT for 2 h, ADP-ribosylated Gαi was detected by immunoblotting using an antibody recognizing mono-ADP-ribose. This revealed that NSC228155 but not NSC29193 prevented ADP-ribosylation of Gαi by PTS1 and, therefore, protected cells from PT-intoxication. Lack of inhibition by NSC29193 might be due to cell impermeability. However, no published data are available on cell permeability of NSC29193 [52]. It was shown before that NSC228155 permeates MDA-MB-468 breast cancer cells and is detected in the cytoplasm, as well as in the nucleus [52,88]. An effect of NSC228155 on cell binding of PT and proteolytic processing of PTS1 was excluded, suggesting that inhibition of enzyme activity by NSC228155 is the underlying mechanism of protection from PT also in living cells.

Most small molecules are able to effectively penetrate tissues and cells which enables them to reach targets such as toxin subunits residing in the cytosol. Thereby, toxin subunits can be inactivated even if they already entered the target cell cytosol. Moreover, production of small molecule inhibitors is relatively easy, cost-effective and not limited to parenteral application compared to other inhibitors such as antibodies. Low specificity and off-target effects represent disadvantages of small molecules [89]. For NSC228155, cytotoxic effects in higher concentrations (20 µM) were shown [52]. Such drawbacks might be improved in the future by structure-based rational drug design approaches available to optimize the small molecule inhibitors.

### 3.4. Neutralizing Antibodies

Antibodies directed against PT can neutralize the unbound toxin (Figure 2). The group of Maynard reported, in 2015, on two murine monoclonal antibodies 1B7 and 11E6 neutralizing PT [53]. These antibodies have been humanized resulting in hu1B7 and hu11E6. Both antibodies retained their low nanomolar affinities for PT. Hu1B7 recognizes an epitope in the B subunit of PT whereas hu11E6 is directed against PTS1 [90,91]. The prophylactically administered binary antibody cocktail protected mice from increased levels of white blood cell counts and decreased bacterial colonization upon infection with *B. pertussis* [53]. An attenuated increase in white blood cell counts due to antibody treatment at day 3 after infection was also observed in adolescent baboons. Additionally, this was accompanied by faster clearance of *B. pertussis* in antibody-treated baboons [53]. The baboon model is an important new animal model for pertussis since it recapitulates the spectrum of human pertussis disease nearly completely. For example, in infected baboons paroxysmal coughing, as well as airborne transmission of *B. pertussis* from animal to animal, were observed [92].

It was shown that neither of the antibodies directly bound *B. pertussis* bacteria or had a supporting effect on the antibody-dependent complement cytotoxicity [56]. Competitive inhibition of PT binding to its cellular receptor was identified as the main mechanism of inhibition for hu11E6. This was shown in vitro by ELISA using fetuin as a model of a terminally sialylated receptor for PT [56]. Hu11E6 inhibited binding of PT to fetuin when present in molar ratios over one. Since hu1B7 only showed a partial inhibition of fetuin binding, the effect of hu1B7 on PT uptake in cells was investigated. Both antibodies were able to bind to receptor-bound PT. However, quantitative immunofluorescence microscopy data suggested that in the presence of hu11E6, PT signals were very low and restricted to the membrane surface instead of being colocalized with organelles associated with the retrograde transport such as the trans Golgi network. This further supports the mechanism of effectively blocking receptor-binding of PT. Pre-equilibration of PT with a 10,000-fold molar excess of hu1B7 did not result in a decreased signal for PT such as with hu11E6 but to reduced co-localization of PT signals with markers of early endosomes, Golgi and ER. In the presence of hu1B7, PT signals were localized primarily near the cell surface. Therefore, in addition to reducing receptor-binding, the data suggest that hu1B7 traps PT at or near the cell surface. This might be due to interfering with internalization or early steps of retrograde trafficking by hu1B7 [56].

Based on the humanized antibodies hu1B7 and hu11E6 recognizing two distinct epitopes of PT, a bispecific antibody was developed, thereby combining the specificity of these antibodies [55]. Binding of the bispecific antibody to PT was similar to the antibody mixture. Comparable synergistic effects were observed for the bispecific antibody and the antibody mixture on inhibiting the PT-induced leukocytosis in a murine model [55].

The antibody hu1B7 was further characterized, optimized and tested for its potential to protect baboons from pertussis if administered prophylactically [54]. The variant hu1B7-YTE was generated to extend the half-life of the antibody by increasing antibody recycling to the serum due to inducing three amino acid changes. Hu1B7 and hu1B7-YTE were administered intravenously to neonatal baboons on day 2 of life and antibody pharmacokinetics were monitored. This experimental design is very similar to the strategy of maternal vaccination which currently represents the most effective concept to prevent severe pertussis in young infants [22]. Vaccination of women in the second or third trimester of pregnancy results in transfer of maternal antibodies onto the fetus and thereby in protection of newborns from pertussis [22,93]. After 5 weeks, baboons were challenged with *B. pertussis* and followed for 6 weeks. A half-life of ~12 days was measured for hu1B7 and ~20 days for hu1B7-YTE resulting in higher serum titers for hu1B7-YTE at the time of infection [54]. Three to four days after infection animals of all treatment groups were comparably colonized by *B. pertussis*. All control animals developed significant leukocytosis and had to be euthanized whereas all antibody-treated animals survived with significantly lower and delayed increase in white blood cell counts compared to controls. Antibody-treated animals also showed reduced symptoms such as coughing and lethargy. Correlating antibody titers with white blood cells counts suggests that PT-neutralizing antibodies are needed at all times during colonization with *B. pertussis* to fully protect against severe symptoms such as leukocytosis. The authors also showed in a mouse model that treatment with anti-PT antibodies did not interfere subsequent acellular PT vaccination [54].

Antibodies provide the advantage of high specificity, high affinity, as well as good tolerability [89]. In context of increasing antibiotic resistance, antibodies targeting disease-causing toxins represent a promising strategy. Low tissue penetration and cell permeability are common disadvantages. In the future, antibody engineering can help to increase efficacy or to overcome drawbacks such as high production costs.

## 4. Conclusions

Although pertussis vaccination rates are high, increasing case numbers have been reported in Western countries, reaching all-time highs since introduction of the first whole cell pertussis vaccine in the 1950s. This is most likely due to reduced effectivity of the acellular vaccine currently used in most high-income countries that only protects against pertussis disease but does not prevent infection or transmission. Fast-waning immunity and genetic adaptation also contribute to the increasing pertussis incidence. In addition, parental vaccine hesitancy can contribute to a greater risk for vaccine-preventable diseases including pertussis [94].

Pertussis is a toxin-mediated disease with PT presenting a main virulence factor to elicit the full spectrum of symptoms including life-threatening leukocytosis. Especially newborns and infants too young to be protected by vaccination are vulnerable to a life-threatening course of disease. Since the only therapeutic intervention available is antibiotic treatment which prevents further transmission but has no effect on pertussis symptoms, novel strategies are needed that target the toxin causing the disease.

In this review, novel inhibitors of PT are presented and summarized in Table 1. These novel inhibitors comprise chaperone inhibitors, human peptides, small molecules inhibiting PT enzyme activity and neutralizing antibodies which all have different advantages and disadvantages. Chaperone inhibitors interfere with the uptake of PTS1 into the cytosol of target cells and can therefore inhibit toxin molecules even if they are already endocytosed. Especially non-immunosuppressive derivatives of already licensed inhibitors such as CsA are promising for extending the therapeutic options to treat pertussis. The recently identified human peptides of the defensin family as inhibitors of PT also provide a new starting point and also contribute to a better understanding of innate immune defense mechanisms against PT. The defensins have to be further characterized in more complex models such as the neonatal mouse infection model regarding their potential to protect infected mice from severe symptoms such as leukocytosis. Small molecules inhibiting PT enzyme activity also bear the advantage to inactivate the toxin even if it already entered the cytosol of cells. The cell-permeable molecule NSC228155 probably binds to the NAD^+^ binding pocket of PTS1 and drawbacks such as cytotoxic effects in higher concentrations can be addressed by structure-based rational drug design approaches. The humanized antibody hu1B7 can bind to cell-bound PT and interferes with endocytosis or other early steps of retrograde transport of PT in the cells. This antibody was already investigated in a baboon infection model using adolescent, as well as neonatal animals, demonstrating a protective effect against pertussis symptoms and lethal course of disease.

Future studies should aim to characterize the mode of action of PT inhibitors in more detail, as well as to optimize inhibitors, for example, regarding their efficacy, cytotoxicity, and route of administration. Since the described inhibitors display different modes of inhibition, a combination of inhibitors targeting the toxin before and after entering target cells might therefore have a beneficial effect.

## Figures and Tables

**Table 1:** Overview of novel strategies inhibiting PT.

Type of Inhibitor & Mechanism	Substances	Models	References
Chaperone inhibitors: Chaperones are required for uptake of PTS1 into target cell cytosol Chaperone inhibition results in reduction of PTS1 in cytosol and protection of cells from intoxication	Radicicol (Hsp90 inhibitor) VER/HA9 (Hsp70 inhibitor) CsA, VK112, NIM811 (Cyp inhibitors) FK506 (FKBP inhibitor)	In vitro (cell-free) CHO cells iGIST bioassay Infant mouse model of pertussis	[41,42,43]
Human peptides: Inhibit enzyme activity of PTS1 in vitro Inhibit intoxication of CHO cells Impair effect of PT on cAMP signaling	α-defensin-1 α-defensin-5 (no inhibition: ß-defensin-1)	In vitro (cell-free) CHO cells iGIST bioassay	[51]
Small molecule inhibitors: Inhibits enzyme activity of PTS1 in vitro Inhibits intoxication of HEK293T cells Binding into NAD^+^ binding pocket of PTS1	NSC228155	In vitro (cell-free) HEK293T cells	[52]
Antibodies: Humanized antibodies targeting PT Hu11E6 recognizes epitope on PTS1 and inhibits binding of PT to cells Hu1B7 recognizes epitope on B-oligomer and interferes with PT-internalization or early steps of retrograde trafficking	Hu1B7 Hu11E6	In vitro (cell-free) CHO cells Mouse infection model Baboon infection model (adolescent and neonatal)	[53,54,55,56]

## Data Availability

Not applicable.

## References

[1] Kilgore P.E., Salim A.M., Zervos M.J., Schmitt H.-J. (2016). Pertussis: Microbiology, Disease, Treatment, and Prevention. Clin. Microbiol. Rev..

[2] Paddock C.D., Sanden G.N., Cherry J.D., Gal A.A., Langston C., Tatti K.M., Wu K.-H., Goldsmith C.S., Greer P.W., Montague J.L. (2008). Pathology and Pathogenesis of Fatal Bordetella Pertussis Infection in Infants. Clin. Infect. Dis..

[3] Zimmerman L.I., Papin J.F., Warfel J., Wolf R.F., Kosanke S.D., Merkel T.J. (2018). Histopathology of Bordetella Pertussis in the Baboon Model. Infect. Immun..

[4] Mattoo S., Cherry J.D. (2005). Molecular Pathogenesis, Epidemiology, and Clinical Manifestations of Respiratory Infections Due to *Bordetella Pertussis* and Other *Bordetella* Subspecies. Clin. Microbiol. Rev..

[5] (2018). Robert-Koch-Institut Infektionsepidemiologisches Jahrbuch Meldepflichtiger Krankheiten für 2018.

[6] Yeung K.H.T., Duclos P., Nelson E.A.S., Hutubessy R.C.W. (2017). An Update of the Global Burden of Pertussis in Children Younger than 5 Years: A Modelling Study. Lancet Infect. Dis..

[7] De Graaf H., Ibrahim M., Hill A.R., Gbesemete D., Vaughan A.T., Gorringe A., Preston A., Buisman A.M., Faust S.N., Kester K.E. (2020). Controlled Human Infection With Bordetella Pertussis Induces Asymptomatic, Immunizing Colonization. Clin. Infect. Dis..

[8] Fedele G., Schiavoni I., Adkins I., Klimova N., Sebo P. (2017). Invasion of Dendritic Cells, Macrophages and Neutrophils by the *Bordetella* Adenylate Cyclase Toxin: A Subversive Move to Fool Host Immunity. Toxins.

[9] Hewlett E., Wolff J. (1976). Soluble Adenylate Cyclase from the Culture Medium of *Bordetella Pertussis*: Purification and Characterization. J. Bacteriol..

[10] Carbonetti N.H. (2015). Contribution of Pertussis Toxin to the Pathogenesis of Pertussis Disease. Pathog. Dis..

[11] Cherry J.D. (2015). The History of Pertussis (Whooping Cough); 1906–2015: Facts, Myths, and Misconceptions. Curr. Epidemiol. Rep..

[12] Connelly C.E., Sun Y., Carbonetti N.H. (2012). Pertussis Toxin Exacerbates and Prolongs Airway Inflammatory Responses during *Bordetella Pertussis* Infection. Infect. Immun..

[13] Scanlon K., Skerry C., Carbonetti N. (2019). Association of Pertussis Toxin with Severe Pertussis Disease. Toxins.

[14] Belcher T., Dubois V., Rivera-Millot A., Locht C., Jacob-Dubuisson F. (2021). Pathogenicity and Virulence of *Bordetella Pertussis* and Its Adaptation to Its Strictly Human Host. Virulence.

[15] Bouchez V., Brun D., Cantinelli T., Dore G., Njamkepo E., Guiso N. (2009). First Report and Detailed Characterization of *B. Pertussis* Isolates Not Expressing Pertussis Toxin or Pertactin. Vaccine.

[16] Scanlon K.M., Chen L., Carbonetti N.H. (2021). Pertussis Toxin Promotes Pulmonary Hypertension in an Infant Mouse Model of *Bordetella Pertussis* Infection. J. Infect. Dis..

[17] Robert-Koch-Institut (2019). Impfquoten bei der Schuleingangsuntersuchung in Deutschland 2017. Epid. Bull..

[18] WHO Immunization Coverage. https://www.who.int/news-room/fact-sheets/detail/immunization-coverage.

[19] Domenech de Cellès M., Magpantay F.M.G., King A.A., Rohani P. (2016). The Pertussis Enigma: Reconciling Epidemiology, Immunology and Evolution. Proc. Biol. Sci..

[20] Locht C., Antoine R. (2021). The History of Pertussis Toxin. Toxins.

[21] Althouse B.M., Scarpino S.V. (2015). Asymptomatic Transmission and the Resurgence of Bordetella Pertussis. BMC Med..

[22] Locht C. (2021). The Path to New Pediatric Vaccines against Pertussis. Vaccines.

[23] Wilkinson K., Righolt C.H., Elliott L.J., Fanella S., Mahmud S.M. (2021). Pertussis Vaccine Effectiveness and Duration of Protection—A Systematic Review and Meta-Analysis. Vaccine.

[24] Esposito S., Stefanelli P., Fry N.K., Fedele G., He Q., Paterson P., Tan T., Knuf M., Rodrigo C., Weil Olivier C. (2019). Pertussis Prevention: Reasons for Resurgence, and Differences in the Current Acellular Pertussis Vaccines. Front. Immunol..

[25] Pittman M. (1984). The Concept of Pertussis as a Toxin-Mediated Disease. Pediatr. Infect. Dis..

[26] Stein P.E., Boodhoo A., Armstrong G.D., Cockle S.A., Klein M.H., Read R.J. (1994). The Crystal Structure of Pertussis Toxin. Structure.

[27] Tamura M., Nogimori K., Murai S., Yajima M., Ito K., Katada T., Ui M., Ishii S. (1982). Subunit Structure of Islet-Activating Protein, Pertussis Toxin, in Conformity with the A-B Model. Biochemistry.

[28] Locht C., Coutte L., Mielcarek N. (2011). The Ins and Outs of Pertussis Toxin. FEBS J..

[29] Armstrong G.D., Howard L.A., Peppler M.S. (1988). Use of Glycosyltransferases to Restore Pertussis Toxin Receptor Activity to Asialoagalactofetuin. J. Biol. Chem..

[30] Hausman S.Z., Burns D.L. (1993). Binding of Pertussis Toxin to Lipid Vesicles Containing Glycolipids. Infect. Immun..

[31] Witvliet M.H., Burns D.L., Brennan M.J., Poolman J.T., Manclark C.R. (1989). Binding of Pertussis Toxin to Eucaryotic Cells and Glycoproteins. Infect. Immun..

[32] Plaut R.D., Carbonetti N.H. (2008). Retrograde Transport of Pertussis Toxin in the Mammalian Cell. Cell. Microbiol..

[33] Teter K. (2019). Intracellular Trafficking and Translocation of Pertussis Toxin. Toxins.

[34] Burns D.L., Manclark C.R. (1986). Adenine Nucleotides Promote Dissociation of Pertussis Toxin Subunits. J. Biol. Chem..

[35] Hazes B., Boodhoo A., Cockle S.A., Read R.J. (1996). Crystal Structure of the Pertussis Toxin-ATP Complex: A Molecular Sensor. J. Mol. Biol..

[36] Plaut R.D., Scanlon K.M., Taylor M., Teter K., Carbonetti N.H. (2016). Intracellular Disassembly and Activity of Pertussis Toxin Require Interaction with ATP. Pathog. Dis..

[37] Banerjee T., Cilenti L., Taylor M., Showman A., Tatulian S.A., Teter K. (2016). Thermal Unfolding of the Pertussis Toxin S1 Subunit Facilitates Toxin Translocation to the Cytosol by the Mechanism of Endoplasmic Reticulum-Associated Degradation. Infect. Immun..

[38] Hazes B., Read R.J. (1997). Accumulating Evidence Suggests That Several AB-Toxins Subvert the Endoplasmic Reticulum-Associated Protein Degradation Pathway to Enter Target Cells. Biochemistry.

[39] Pande A.H., Moe D., Jamnadas M., Tatulian S.A., Teter K. (2006). The Pertussis Toxin S1 Subunit Is a Thermally Unstable Protein Susceptible to Degradation by the 20S Proteasome. Biochemistry.

[40] Worthington Z.E.V., Carbonetti N.H. (2007). Evading the Proteasome: Absence of Lysine Residues Contributes to Pertussis Toxin Activity by Evasion of Proteasome Degradation. Infect. Immun..

[41] Ernst K., Mittler A.-K., Winkelmann V., Kling C., Eberhardt N., Anastasia A., Sonnabend M., Lochbaum R., Wirsching J., Sakari M. (2021). Pharmacological Targeting of Host Chaperones Protects from Pertussis Toxin in Vitro and in Vivo. Sci. Rep..

[42] Ernst K., Eberhardt N., Mittler A.-K., Sonnabend M., Anastasia A., Freisinger S., Schiene-Fischer C., Malešević M., Barth H. (2018). Pharmacological Cyclophilin Inhibitors Prevent Intoxication of Mammalian Cells with Bordetella Pertussis Toxin. Toxins.

[43] Kellner A., Taylor M., Banerjee T., Britt C.B.T., Teter K. (2019). A Binding Motif for Hsp90 in the A Chains of ADP-Ribosylating Toxins That Move from the Endoplasmic Reticulum to the Cytosol. Cell. Microbiol..

[44] Bokoch G.M., Katada T., Northup J.K., Hewlett E.L., Gilman A.G. (1983). Identification of the Predominant Substrate for ADP-Ribosylation by Islet Activating Protein. J. Biol. Chem..

[45] Katada T., Ui M. (1982). Direct Modification of the Membrane Adenylate Cyclase System by Islet-Activating Protein Due to ADP-Ribosylation of a Membrane Protein. Proc. Natl. Acad. Sci. USA.

[46] Andreasen C., Carbonetti N.H. (2008). Pertussis Toxin Inhibits Early Chemokine Production to Delay Neutrophil Recruitment in Response to *Bordetella Pertussis* Respiratory Tract Infection in Mice. Infect. Immun..

[47] Kirimanjeswara G.S., Agosto L.M., Kennett M.J., Bjornstad O.N., Harvill E.T. (2005). Pertussis Toxin Inhibits Neutrophil Recruitment to Delay Antibody-Mediated Clearance of *Bordetella Pertussis*. J. Clin. Investig..

[48] Spangrude G.J., Sacchi F., Hill H.R., Van Epps D.E., Daynes R.A. (1985). Inhibition of Lymphocyte and Neutrophil Chemotaxis by Pertussis Toxin. J. Immunol..

[49] Carbonetti N.H. (2016). *Bordetella pertussis*: New Concepts in Pathogenesis and Treatment. Curr. Opin. Infect. Dis..

[50] Altunaiji S., Kukuruzovic R., Curtis N., Massie J. (2007). Antibiotics for Whooping Cough (Pertussis). Cochrane Database Syst. Rev..

[51] Kling C., Pulliainen A.T., Barth H., Ernst K. (2021). Human Peptides α-Defensin-1 and -5 Inhibit Pertussis Toxin. Toxins.

[52] Ashok Y., Miettinen M., de Oliveira D.K.H., Tamirat M.Z., Näreoja K., Tiwari A., Hottiger M.O., Johnson M.S., Lehtiö L., Pulliainen A.T. (2020). Discovery of Compounds Inhibiting the ADP-Ribosyltransferase Activity of Pertussis Toxin. ACS Infect. Dis..

[53] Nguyen A.W., Wagner E.K., Laber J.R., Goodfield L.L., Smallridge W.E., Harvill E.T., Papin J.F., Wolf R.F., Padlan E.A., Bristol A. (2015). A Cocktail of Humanized Anti-Pertussis Toxin Antibodies Limits Disease in Murine and Baboon Models of Whooping Cough. Sci. Transl. Med..

[54] Nguyen A.W., DiVenere A.M., Papin J.F., Connelly S., Kaleko M., Maynard J.A. (2020). Neutralization of Pertussis Toxin by a Single Antibody Prevents Clinical Pertussis in Neonatal Baboons. Sci. Adv..

[55] Wagner E.K., Wang X., Bui A., Maynard J.A. (2016). Synergistic Neutralization of Pertussis Toxin by a Bispecific Antibody In Vitro and In Vivo. Clin. Vaccine Immunol..

[56] Acquaye-Seedah E., Huang Y., Sutherland J.N., DiVenere A.M., Maynard J.A. (2018). Humanised Monoclonal Antibodies Neutralise Pertussis Toxin by Receptor Blockade and Reduced Retrograde Trafficking. Cell. Microbiol..

[57] Ernst K., Sailer J., Braune M., Barth H. (2021). Intoxication of Mammalian Cells with Binary Clostridial Enterotoxins Is Inhibited by the Combination of Pharmacological Chaperone Inhibitors. Naunyn Schmiedebergs Arch. Pharmacol..

[58] Ernst K., Schnell L., Barth H. (2017). Host Cell Chaperones Hsp70/Hsp90 and Peptidyl-Prolyl Cis/Trans Isomerases Are Required for the Membrane Translocation of Bacterial ADP-Ribosylating Toxins. Curr. Top. Microbiol. Immunol..

[59] Moss J., Stanley S.J., Watkins P.A., Burns D.L., Manclark C.R., Kaslow H.R., Hewlett E.L. (1986). Stimulation of the Thiol-Dependent ADP-Ribosyltransferase and NAD Glycohydrolase Activities of Bordetella Pertussis Toxin by Adenine Nucleotides, Phospholipids, and Detergents. Biochemistry.

[60] Hausman S.Z., Manclark C.R., Burns D.L. (1990). Binding of ATP by Pertussis Toxin and Isolated Toxin Subunits. Biochemistry.

[61] Barth H., Ernst K., Gopalakrishnakone P., Stiles B., Alape-Girón A., Dubreuil J.D., Mandal M. (2016). Chaperones and ADP-Ribosylating Bacterial Toxins. Microbial Toxins.

[62] Ernst K., Kling C., Landenberger M., Barth H. (2018). Combined Pharmacological Inhibition of Cyclophilins, FK506-Binding Proteins, Hsp90, and Hsp70 Protects Cells From Clostridium Botulinum C2 Toxin. Front. Pharmacol..

[63] Ernst K., Liebscher M., Mathea S., Granzhan A., Schmid J., Popoff M.R., Ihmels H., Barth H., Schiene-Fischer C. (2016). A Novel Hsp70 Inhibitor Prevents Cell Intoxication with the Actin ADP-Ribosylating Clostridium Perfringens Iota Toxin. Sci. Rep..

[64] Kaiser E., Böhm N., Ernst K., Langer S., Schwan C., Aktories K., Popoff M., Fischer G., Barth H. (2012). FK506-Binding Protein 51 Interacts with *Clostridium Botulinum* C2 Toxin and FK506 Inhibits Membrane Translocation of the Toxin in Mammalian Cells. Cell. Microbiol..

[65] Lang A.E., Ernst K., Lee H., Papatheodorou P., Schwan C., Barth H., Aktories K. (2014). The Chaperone Hsp90 and PPIases of the Cyclophilin and FKBP Families Facilitate Membrane Translocation of Photorhabdus Luminescens ADP-Ribosyltransferases. Cell. Microbiol..

[66] Biebl M.M., Buchner J. (2019). Structure, Function, and Regulation of the Hsp90 Machinery. Cold Spring Harb. Perspect. Biol..

[67] Li J., Soroka J., Buchner J. (2012). The Hsp90 Chaperone Machinery: Conformational Dynamics and Regulation by Co-Chaperones. Biochim. Biophys. Acta.

[68] Williamson D.S., Borgognoni J., Clay A., Daniels Z., Dokurno P., Drysdale M.J., Foloppe N., Francis G.L., Graham C.J., Howes R. (2009). Novel Adenosine-Derived Inhibitors of 70 KDa Heat Shock Protein, Discovered through Structure-Based Design. J. Med. Chem..

[69] Fischer G., Schmid F.-X. (2001). Peptidylproline Cis–Trans-Isomerases. eLS.

[70] Galat A. (2003). Peptidylprolyl Cis/Trans Isomerases (Immunophilins): Biological Diversity—Targets—Functions. Curr. Top. Med. Chem..

[71] Gray M.C., Guerrant R.L., Hewlett E.L. (2021). The CHO Cell Clustering Response to Pertussis Toxin: History of Its Discovery and Recent Developments in Its Use. Toxins.

[72] Hewlett E.L., Sauer K.T., Myers G.A., Cowell J.L., Guerrant R.L. (1983). Induction of a Novel Morphological Response in Chinese Hamster Ovary Cells by Pertussis Toxin. Infect. Immun..

[73] Ernst K., Langer S., Kaiser E., Osseforth C., Michaelis J., Popoff M.R., Schwan C., Aktories K., Kahlert V., Malesevic M. (2015). Cyclophilin-Facilitated Membrane Translocation as Pharmacological Target to Prevent Intoxication of Mammalian Cells by Binary Clostridial Actin ADP-Ribosylated Toxins. J. Mol. Biol..

[74] Ernst K., Schmid J., Beck M., Hägele M., Hohwieler M., Hauff P., Ückert A.K., Anastasia A., Fauler M., Jank T. (2017). Hsp70 Facilitates Trans-Membrane Transport of Bacterial ADP-Ribosylating Toxins into the Cytosol of Mammalian Cells. Sci. Rep..

[75] Kaiser E., Kroll C., Ernst K., Schwan C., Popoff M., Fischer G., Buchner J., Aktories K., Barth H. (2011). Membrane Translocation of Binary Actin-ADP-Ribosylating Toxins from Clostridium Difficile and Clostridium Perfringens Is Facilitated by Cyclophilin A and Hsp90. Infect. Immun..

[76] Scanlon K.M., Snyder Y.G., Skerry C., Carbonetti N.H. (2017). Fatal Pertussis in the Neonatal Mouse Model Is Associated with Pertussis Toxin-Mediated Pathology beyond the Airways. Infect. Immun..

[77] Gestal M.C., Johnson H.M., Harvill E.T. (2019). Immunomodulation as a Novel Strategy for Prevention and Treatment of *Bordetella* Spp. Infections. Front. Immunol..

[78] Cederlund A., Gudmundsson G.H., Agerberth B. (2011). Antimicrobial Peptides Important in Innate Immunity. FEBS J..

[79] Zhao L., Lu W. (2014). Defensins in Innate Immunity. Curr. Opin. Hematol..

[80] Fischer S., Ückert A.-K., Landenberger M., Papatheodorou P., Hoffmann-Richter C., Mittler A.-K., Ziener U., Hägele M., Schwan C., Müller M. (2020). Human Peptide α-Defensin-1 Interferes with *Clostridioides Difficile* Toxins TcdA, TcdB, and CDT. FASEB J..

[81] Giesemann T., Guttenberg G., Aktories K. (2008). Human α-Defensins Inhibit *Clostridium Difficile* Toxin B. Gastroenterology.

[82] Kim C., Gajendran N., Mittrücker H.-W., Weiwad M., Song Y.-H., Hurwitz R., Wilmanns M., Fischer G., Kaufmann S.H.E. (2005). Human α-Defensins Neutralize Anthrax Lethal Toxin and Protect against Its Fatal Consequences. Proc. Natl. Acad. Sci. USA.

[83] Korbmacher M., Fischer S., Landenberger M., Papatheodorou P., Aktories K., Barth H. (2020). Human α-Defensin-5 Efficiently Neutralizes *Clostridioides Difficile* Toxins TcdA, TcdB, and CDT. Front. Pharmacol..

[84] Kudryashova E., Seveau S.M., Kudryashov D.S. (2017). Targeting and Inactivation of Bacterial Toxins by Human Defensins. Biol. Chem..

[85] Kudryashova E., Quintyn R., Seveau S., Lu W., Wysocki V.H., Kudryashov D.S. (2014). Human Defensins Facilitate Local Unfolding of Thermodynamically Unstable Regions of Bacterial Protein Toxins. Immunity.

[86] Paramonov V.M., Sahlgren C., Rivero-Müller A., Pulliainen A.T. (2020). IGIST-A Kinetic Bioassay for Pertussis Toxin Based on Its Effect on Inhibitory GPCR Signaling. ACS Sens..

[87] Lee A.C.-L., Harris J.L., Khanna K.K., Hong J.-H. (2019). A Comprehensive Review on Current Advances in Peptide Drug Development and Design. Int. J. Mol. Sci..

[88] Sakanyan V., Hulin P., Alves de Sousa R., Silva V.A.O., Hambardzumyan A., Nedellec S., Tomasoni C., Logé C., Pineau C., Roussakis C. (2016). Activation of EGFR by Small Compounds through Coupling the Generation of Hydrogen Peroxide to Stable Dimerization of Cu/Zn SOD1. Sci. Rep..

[89] Sakari M., Laisi A., Pulliainen A.T. (2022). Exotoxin-Targeted Drug Modalities as Antibiotic Alternatives. ACS Infect. Dis..

[90] Sutherland J.N., Maynard J.A. (2009). Characterization of a Key Neutralizing Epitope on Pertussis Toxin Recognized by the Monoclonal Antibody 1B7. Biochemistry.

[91] Sato H., Sato Y., Ito A., Ohishi I. (1987). Effect of Monoclonal Antibody to Pertussis Toxin on Toxin Activity. Infect. Immun.

[92] Pinto M.V., Merkel T.J. (2017). Pertussis Disease and Transmission and Host Responses: Insights from the Baboon Model of Pertussis. J. Infect..

[93] Maertens K., Orije M.R.P., Van Damme P., Leuridan E. (2020). Vaccination during Pregnancy: Current and Possible Future Recommendations. Eur. J. Pediatr..

[94] Nguyen K.H., Srivastav A., Lindley M.C., Fisher A., Kim D., Greby S.M., Lee J., Singleton J.A. (2022). Parental Vaccine Hesitancy and Association with Childhood Diphtheria, Tetanus Toxoid, and Acellular Pertussis; Measles, Mumps, and Rubella; Rotavirus; and Combined 7-Series Vaccination. Am. J. Prev. Med..

